# Short-Term Striatal Gene Expression Responses to Brain-Derived Neurotrophic Factor Are Dependent on MEK and ERK Activation

**DOI:** 10.1371/journal.pone.0005292

**Published:** 2009-04-23

**Authors:** Ozgun Gokce, Heike Runne, Alexandre Kuhn, Ruth Luthi-Carter

**Affiliations:** Laboratory of Functional Neurogenomics, Brain Mind Institute, École Polytechnique Fédérale de Lausanne (EPFL), Lausanne, Switzerland; University of North Dakota, United States of America

## Abstract

**Background:**

Brain-derived neurotrophic factor (BDNF) is believed to be an important regulator of striatal neuron survival, differentiation, and plasticity. Moreover, reduction of BDNF delivery to the striatum has been implicated in the pathophysiology of Huntington's disease. Nevertheless, many essential aspects of BDNF responses in striatal neurons remain to be elucidated.

**Methodology/Principal Findings:**

In this study, we assessed the relative contributions of multipartite intracellular signaling pathways to the short-term induction of striatal gene expression by BDNF. To identify genes regulated by BDNF in these GABAergic cells, we first used DNA microarrays to quantify their transcriptomic responses following 3 h of BDNF exposure. The signal transduction pathways underlying gene induction were subsequently dissected using pharmacological agents and quantitative real-time PCR. Gene expression responses to BDNF were abolished by inhibitors of TrkB (K252a) and calcium (chelator BAPTA-AM and transient receptor potential cation channel [TRPC] antagonist SKF-96365). Interestingly, inhibitors of mitogen-activated protein kinase kinases 1 and 2 (MEK1/2) and extracellular signal-regulated kinase ERK also blocked the BDNF-mediated induction of all tested BDNF-responsive genes. In contrast, inhibitors of nitric oxide synthase (NOS), phosphotidylinositol-3-kinase (PI3K), and CAMK exhibited less prevalent, gene-specific effects on BDNF-induced RNA expression. At the nuclear level, the activation of both Elk-1 and CREB showed MEK dependence. Importantly, MEK-dependent activation of transcription was shown to be required for BDNF-induced striatal neurite outgrowth, providing evidence for its contribution to striatal neuron plasticity.

**Conclusions:**

These results show that the MEK/ERK pathway is a major mediator of neuronal plasticity and other important BDNF-dependent striatal functions that are fulfilled through the positive regulation of gene expression.

## Introduction

Medium-sized spiny striatal neurons (MSNs) comprise >90% of all striatal neurons and serve the functions of integrating and transmitting information from the cerebral cortex to the output nuclei of the basal ganglia [1], [2], [3]. This circuit is crucial for many important functions, such as the regulation of voluntary movement, and is also involved in human neurodegenerative and neuropsychiatric disorders. MSNs are GABAergic and provide inhibitory input from the striatum to the globus pallidus and substantia nigra pars reticulata.

The striatum is highly innervated by BDNF-releasing synapses. BDNF is delivered to the striatum via activity-dependent anterograde release from excitatory corticostriatal axons [4], [5]. The importance of BDNF regulation of striatal function is exemplified by its enhancement of survival and morphological and biochemical differentiation of striatal neurons *in vitro* [6], [7] and by the phenotype of forebrain-specific BDNF-knock-out mice (Emx-BDNF, [8]) which have diminished striatal volumes and exhibit behavioural abnormalities characteristic of striatal dysfunction.

**Figure 1:**
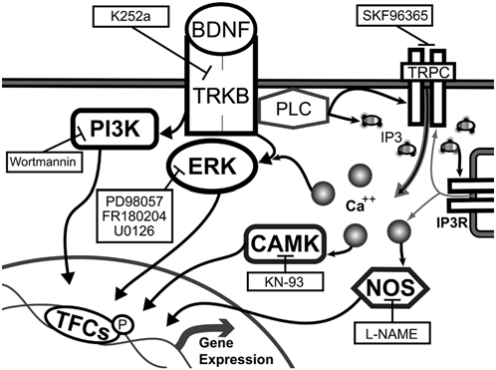
Schematic representation of possible signaling pathways by which BDNF regulates mRNA expression in striatal neurons. Shown are known mechanisms by which BDNF-regulated signaling has been previously implicated in regulating gene expression in other cell types. Four main arms of TrkB-dependent signaling are identified involving PI3K, ERK, CAMK, and NOS (see text). Pharmacologic agents used in the present study to assess the contributions of individual branches or molecular entities of these pathways are indicated. TFCs designates transription factor complexes, including those containing SRF and TCF/Elk-1, which bind to serum response elements (SREs) in target gene promoters to mediate transcriptional activation, and CBP and CREB, which enhance transcription via association with cAMP-response element (CRE) motifs.

BDNF signaling is mediated primarily by its high affinity receptor, the receptor tyrosine kinase tropomyosin-related kinase B (TrkB) (Fig. 1). TrkB activation initiates three major intracellular signaling cascades: the shc/Frs2 and ras/raf-mediated activation of a MAPK phosphorylation cascade involving MEK and ERK, the shc/Frs2 and GAB1-mediated activation of a phoshphotidylinositol-3-kinase PI3K pathway involving Akt1, and the direct TrkB-mediated activation of phoshpolipase C gamma (PLCγ) which produces inositol triphosphate (IP3) and diacylglycerol (DAG) and increases intracellular free calcium [9], [10]. BDNF-induced calcium mobilization has also been shown in some cases to be coupled to calcium entry via transient receptor potential cation channels (TRPCs) [11], [12]. Downstream calcium-dependent responses to BDNF involve the activation of calcium-calmodulin-dependent kinases (CAMKs) [13], [14] and nitric oxide synthase (NOS) [15], [16]. These various signaling cascades have been shown to mediate independent as well as coordinate actions that are responsible for both distinct and overlapping BDNF-dependent functions.

Downstream mechanisms of BDNF signaling include the initiation of gene transcription. Known mediators of BDNF-regulated transcription include the cAMP response element binding protein (CREB) [14], [17] and the ternary complex factor (TFC) protein Elk-1 [18], [19]. The activities of these transcription factors are positively regulated by phosphorylation, which is a major mechanism of their conveying BDNF-stimulated responses. Although previous studies have examined gene regulation by BDNF globally in selected cell types, this important aspect of BDNF signaling has never before been addressed in GABAergic neurons, including those of the striatum. Given the compelling relevance of this question for striatal biology and human nervous system disorders, we set out to determine the overall balance of mechanisms by which BDNF-mediated signaling regulates MSN gene expression. In order to assess which genes were responsive to BDNF in striatal cells, BDNF-regulated RNAs were first determined systematically using high-density microarrays. Subsequently, the signal transduction pathways that underlie these transcriptional responses were dissected using pharmacological agents and quantitative real-time PCR. The results demonstrate that BDNF-induced TrkB activation and calcium entry via TRPC are both required for BDNF transcription effects. Downstream of TrkB, we further show that the MAPK pathway involving MEK/ERK activation is a crucial and universal regulator of BDNF-induced gene expression. In addition, our results suggest that BDNF-induced RNA responses in MSNs are modulated by PI3K, NOS, and CAMK in a gene-specific manner.

## Materials and Methods

### Ethics Statement

All experiments were approved by the local veterinary office and the Commission for Animal Experimentation of the Canton of Vaud Switzerland.

### Preparation of striatal neuron cultures

Dissociated primary striatal neuron cultures were prepared from ganglionic eminences of E16 rat embryos as described in [20]. This procedure has been previously demonstrated to yield a high proportion of neuronal cells expressing MSN markers (*e.g.* 95–98% of NeuN-positive cells express high levels of DARPP-32) and exhibiting an electrophysiologic behavior characteristic of GABAergic neurons [21]. Experimental treatments were performed between 2–4 weeks *in vitro* and at least 3 days after a change of culture medium. BDNF responses were highly consistent between cultures of these ages (and up to 6 weeks *in vitro*, data not shown).

### Pharmacologic treatments

To study the effects of BDNF on gene expression, striatal cultures were treated for 3 hours with 50 ng/mL BDNF (R&D Systems) or vehicle (0.1% BSA in PBS) at 2–4 weeks *in vitro* unless otherwise indicated. For pharmacologic inhibitor studies, cells were treated for 30 min prior to BDNF stimulation with wortmannin (100 nM, Sigma), KN-93 (5 µM, Sigma), PD98059 (50 µM, Sigma), K252a (200 nM, Sigma), L-NAME (2 mM, Sigma), U0126 (30 µM, Sigma), FR180204 (100 µM, Calbiochem), cycloheximide (0.5 ug/ml, Sigma), actinomycin D (2 ug/ml, Sigma), BAPTA-AM (100 µM, Sigma), or SKF-96365 hydrochloride (100 µM, Sigma). For calcium experiments, culture medium was replaced with physiologic solution at pH 7.4 composed of (in mM): 125 NaCl, 25 NaHCO_3_, 25 glucose, 2.5 KCl, 1.25 NaH_2_PO4, 1 MgCl_2_, 2 CaCl_2_ (unless otherwise indicated).

### RNA analyses

RNA was extracted using the RNeasy system (Qiagen). For microarray analyses, one microgram of total RNA of each sample (n = 4) was used to prepare biotinylated fragmented cRNA, which was produced and hybridized to Affymetrix Rat Genome 230 2.0 GeneChip microarrays according to the GeneChip Expression Analysis manual. Gene expression was quantified by robust multi-array analysis [22] using the R software package affy [23]. All statistical analyses of gene expression were carried out with the R software package limma [24] using a false-discovery rate (FDR) approach to correct for multiple testing [25], with a significance threshold of FDR p<0.05.

cDNA preparation for quantitative real-time PCR (QPCR) analysis utilized the High Capacity cDNA RT kit (Applied Biosystems). Samples were analyzed for BDNF induced transcripts using Taqman expression assays (Applied Biosystems) as follows: beta-actin (Actb), Rn00667869_m1; activity regulated cytoskeletal-associated protein (Arc), Rn00571208_g1; brain-specific angiogenesis inhibitor 1-associated protein 2 (Baiap2), Rn00589411_m1; dual specificity phosphatase 6 (Dusp6), Rn00518185_m1; early growth response 1 (Egr1), Rn00561138_m1; early growth response 2 (Egr2), Rn00586224_m1; Kruppel-like factor 5 (Klf5), Rn00821442_g1; Ngfi-A binding protein 2 (Nab2), Rn01505957_g1; prepronociceptin (Pnoc), Rn00564560_m1; protein phosphatase 2C, magnesium dependent catalytic subunit (Ppm2c), Rn00571345_m1; synaptotagmin IV (Syt4), Rn01157571_m1; tachykinin 1 (Tac1), Rn00562002_m1. Gene expression measures were derived from biological triplicates/quadruplicates and technical triplicates performed on a 7900HT Real-Time PCR System with SDS 2.3 software (Applied Biosystems). Relative expression (V) was calculated by normalization to ß-actin expression as described in [26]. Criteria for presentation of specific RNA data were a statistically significant change in expression in BDNF- versus vehicle-treated cells of at least 30% in the corresponding experiment.

### Immunostaining, DAF-FM DA staining, and microscopic image analyses

Cell cultures were washed with cold PBS and fixed with 4% paraformaldehyde (Fluka) for 15 min at 4°C. Cultures were subsequently washed with PBS and incubated in a blocking solution of PBS with 10% normal goat serum (NGS) (Dakocytomation) and 0.1% Triton X-100 (Sigma). Cells were then incubated overnight at 4°C in blocking solution containing primary antibodies: rabbit anti-GFP (Chemicon, 1∶500), rabbit anti-TrkB (Millipore 1∶500) and/or mouse monoclonal anti-Neuronal Nuclei (NeuN) (Chemicon MAB377 clone A60, 1∶400). Secondary antibodies coupled to fluorophores (Alexa Fluor 488 [1∶1,000], goat anti-rabbit IgG Alexa Fluor 488 [1∶500], goat anti-mouse IgG Alexa Fluor 594 [1∶500], all from Invitrogen) were applied for 2 h at room temperature.

For neurite outgrowth analyses, DIV3 primary striatal cultures were transfected with plasmids encoding enhanced green fluorescent protein (EGFP) using NeuroFECT reagent (Genlantis), resulting in sparse and well-separated EGFP-positive neurons. Inhibitor treatments began 30 min before BDNF treatments, and cells were exposed to BDNF for 24 hours. At the end of the BDNF treatment, cells were fixed and labeled with anti-GFP antibodies as described above. Images of EGFP-positive cells were acquired with a Leica DMI 4000 microscope and the length of the longest GFP-positive process of each neuron was measured with ImageJ software [27]. For each condition, averages of 85 neurons were analyzed.

For nitric oxide synthase (NOS) activity measurements, 3 week *in vitro* primary striatal cultures were preincubated with DMEM containing DAF-FM DA (Molecular Probes; 10 µM) for 30 min at 37°C. Selected cultures were also pretreated with the NOS inhibitor L-NAME (2 mM) for 30 min. For each condition a minimum of 4 images from 4 cultures were taken and analyzed. Total NO/DAF-FM DA-related fluorescence intensity was calculated using MetaMorph 7.5 software (Molecular Devices).

### Immunoblotting

For analysis of phosphoproteins, primary striatal cultures were pretreated with pharmacologic inhibitors for 30 min prior to stimulation with BDNF for 15 min. Cells were harvested in RIPA buffer (Sigma), containing 1× protease inhibitor cocktail (Sigma) and 1× phophatase inhibitor cocktails 1 and 2 (Sigma). Proteins were separated on 12.5% SDS-polyacrylamide gels and transferred to nitrocellulose. Membranes were blocked for one hour in 30% Odyssey® Blocking Buffer in PBS (LI-COR Biosciences) followed by incubation in primary antibodies diluted in Blocking Buffer with 0.1% Tween-20 overnight at 4°C. Membranes were rinsed with 0.1% Tween-20/PBS followed by incubation in secondary antibody diluted in Blocking Solution for two hours at RT. After final rinses, blots were scanned with an Odyssey® Infrared Imager and densitometric measurements were obtained using Odyssey software (LI-COR Biosciences). Primary and secondary antibodies comprised: mouse monoclonal anti-phospho-CREB (Ser133) (Upstate clone 634-2, 1∶1,000), rabbit anti-phospho-p44/42 MAPK (Erk1/2) (Thr202/Tyr204) (Cell signaling, 1∶1,000), rabbit anti-phospho-Akt (Ser473) (Cell Signaling, 1∶1,000), mouse monoclonal anti-phospho-Elk-1 (Ser 383) (Santa Cruz, 1∶1,000), goat anti-actin (Santa Cruz, 1∶10,000), mouse monoclonal anti-tubulin (Sigma clone B-5-1-2, 1∶10,000), donkey anti-mouse IRDye 800CW (LI-COR Biosciences, 1∶20,000), donkey anti-rabbit IRDye 680 (LI-COR Biosciences, 1∶20,000), donkey anti-goat IRDye 800CW (LI-COR Biosciences,1∶20,000).

### Enhancer element reporter assays

For CRE activity measurements, a lentiviral vector containing 6 CRE sites fused to a luciferase reporter gene (SIN-6xCRE-luc2CP-WHV, CRE-Luc) was produced in human embryonic kidney 293T (HEK293T) cells with a four-plasmid system as described previously [28]. The virus was resuspended in PBS with 1% BSA and matched for particle content to 1,500 ng of p24 antigen per milliliter as measured by ELISA (RETROtek; Gentaur). Primary striatal cultures were infected at a ratio of 1 ng p24/10,000 cells with CRE-Luc at DIV9. At 2 weeks *in vitro*, selected cultures were pretreated with 50 µM PD98059 for 30 min followed by BDNF treatment (50 ng/ml) for 90 min. Activity was assessed with the Promega Luciferase Assay System according to the supplier's instructions. Luminescence was measured on a TECAN GenioPro plate reader in the linear range of the instrument.

For measurements of SRE activity, the Chroma-Glo Luciferase Assay System (Promega) was used. Primary striatal cultures were co-transfected at 2 weeks *in vitro* (using NeuroFECT reagent, Genlantis) with a plasmid containing 2 SRE enhancer sites and a minimal promoter fused to a green-emitting luciferase (SIN-cPPT-SRE-CBG99-WHV) and a plasmid containing the PGK promoter fused to a red-emitting luciferase (SIN-cPPT-PGK-CBR-WHV), which was used as a control reporter to normalize for transfection efficiency. 36 hours after transfection, selected cultures were pretreated with 50 µM PD98059 for 30 min followed by BDNF treatment (50 ng/ml) for 90 min. Luminescence was measured on a TECAN GenioPro plate reader in the linear range of the instrument.

### Acute dissociation of striatal neurons from adult rat brain

4-week-old Sprague–Dawley rats were sacrificed by CO_2_ inhalation, after which the striata were dissected, minced into small pieces and digested as described previously [29] with the following modifications. Minced striatal tissue was treated with papain (20 U/ml, Sigma) for 30–40 min at 37°C in a gently rotating chamber, followed by repeated pipetting with a fire-polished Pasteur pipette. The resultant cells were then transferred to 12-well tissue culture plates in which they were treated 3 hours later with BDNF in the presence or absence of the MEK inhibitor PD98059.

## Results

### Global analysis of BDNF-induced acute mRNA responses in cultured striatal neurons

In order to achieve a comprehensive measure of acute gene expression responses to BDNF in striatal neurons, we used microarray gene expression profiling to assay global mRNA expression differences following 3 h treatment of primary striatal cultures with BDNF. This time point was chosen in order to detect both immediate early and cumulative acute responses, based on previous analyses of gene expression in other cell types [30] as well as preliminary studies of striatal responses in our laboratory (data not shown). At a cutoff of false-discovery rate-corrected p<0.05 [25], approximately 14% of all microarray probesets (4440/31042) showed differential expression after BDNF treatment, comprising 2359 upregulated and 2081 downregulated RNA probesets (Suppl. Table S1). Overall, BDNF-induced responses showed large gene-dependent variations in fold induction (or repression); known immediate-early response genes such as Egr1, Egr2, and Egr3 showed 15–20-fold changes, whereas the majority of genes at the above cutoff showed a fold-change below 2. Notably, however, restricting the analysis to genes regulated more than 2-fold revealed a higher enrichment of upregulated compared to downregulated RNAs (166 increased, 46 decreased). Selected BDNF-responsive genes showing 2-fold or greater regulation are shown in Table 1.

**Table 1:** Selected genes regulated by BDNF in striatal neurons.

*Probeset ID*	*Gene Symbol*	*Gene Title*	*Log2 FC*	*FDR p value*
1370454_at	Homer1	homer 1	4.79	1.14E-05
1387306_a_at	Egr2	early growth response 2	3.92	1.46E-06
1375043_at	Fos	FBJ murine osteosarcoma viral oncogene homolog	3.90	1.64E-05
1392791_at	Egr3	Early growth response 3	3.61	8.76E-07
1368321_at	Egr1	early growth response 1	3.40	1.21E-05
1394039_at	Klf5	Kruppel-like factor 5	3.22	2.02E-06
1368359_a_at	Vgf	VGF nerve growth factor inducible	3.14	1.75E-04
1377727_at	Baz1a	bromodomain adjacent to zinc finger domain, 1A	3.12	2.95E-06
1372510_at	Srxn1	Sulfiredoxin 1	2.44	1.64E-05
1382521_at	Gls	glutaminase	2.35	7.50E-06
1374925_at	Nab2	Ngfi-A binding protein 2	2.28	7.82E-06
1368369_at	Pnoc	prepronociceptin	2.26	1.51E-06
1368910_at	Ppm2c	protein phosphatase 2C, magnesium dependent, catalytic subunit	2.22	1.72E-06
1373324_at	Dusp14	dual specificity phosphatase 14	2.02	5.06E-07
1368303_at	Per2	period homolog 2 (Drosophila)	1.97	7.82E-06
1393638_at	Ptger4	Prostaglandin E receptor 4 (subtype EP4)	1.87	4.40E-07
1376569_at	Klf2	Kruppel-like factor 2	1.87	1.90E-05
1368650_at	Klf10	Kruppel-like factor 10	1.86	1.84E-05
1372417_at	Sertad1	SERTA domain containing 1	1.83	8.92E-06
1370805_at	Cited1	Cbp/p300-interacting transactivator with Glu/Asp-rich carboxy-terminal domain 1	1.82	4.38E-07
1368124_at	Dusp5	dual specificity phosphatase 5	1.81	1.72E-06
1368106_at	Plk2	polo-like kinase 2 (Drosophila)	1.79	1.66E-05
1378407_at	Trim9	Tripartite motif protein 9	1.78	8.13E-06
1373624_at	Rassf8	Ras association (RalGDS/AF-6) domain family 8	1.78	3.04E-06
1387662_at	Syt4	synaptotagmin IV	1.77	3.42E-05
1372459_at	Vasp	vasodilator-stimulated phosphoprotein	1.76	5.37E-06
1388792_at	Gadd45g	growth arrest and DNA-damage-inducible 45 gamma	1.73	3.04E-06
1372389_at	Ier2	immediate early response 2	1.73	1.72E-06
1378519_at	Cpne1	Copine I	1.71	1.21E-05
1387391_at	Cdkn1a	cyclin-dependent kinase inhibitor 1A	1.70	8.72E-05
1387068_at	Arc	activity regulated cytoskeletal-associated protein	1.66	1.93E-04
1383697_at	Slc5a3	Solute carrier family 5 (inositol transporters), member 3	1.65	1.06E-04
1368947_at	Gadd45a	growth arrest and DNA-damage-inducible 45 alpha	1.58	5.06E-07
1388858_at	Map2k3	mitogen activated protein kinase kinase 3	1.56	5.17E-06
1398483_at	Rgs17	Regulator of G-protein signaling 17	1.56	1.59E-05
1385592_at	Bcor	Bcl6 interacting corepressor	1.49	1.95E-04
1369303_at	Crh	corticotropin releasing hormone	1.48	1.21E-04
1387276_at	Ania4	activity and neurotransmitter-induced early gene protein 4	1.44	2.98E-06
1370074_at	Baiap2	brain-specific angiogenesis inhibitor 1-associated protein 2	1.43	9.61E-06
1373093_at	Errfi1	ERBB receptor feedback inhibitor 1	1.42	1.76E-04
1379375_at	Pdgfa	Platelet derived growth factor, alpha	1.41	2.52E-06
1393172_at	Nab1	Ngfi-A binding protein 1	1.41	2.95E-06
1389263_at	Rai14	retinoic acid induced 14	1.40	2.00E-05
1397363_at	Pvrl3	poliovirus receptor-related 3	1.39	2.30E-05
1378447_at	Thrap1	Thyroid hormone receptor associated protein 1	1.37	2.95E-05
1378081_at	Tgfb1i4	Transforming growth factor beta 1 induced transcript 4	1.31	1.10E-05
1387442_at	Egr4	early growth response 4	1.30	9.98E-04
1387060_at	Klf6	Kruppel-like factor 6	1.28	1.55E-05
1370333_a_at	Igf1	insulin-like growth factor 1	1.27	6.16E-05
1389355_at	Ier5	immediate early response 5	1.23	2.14E-05
1368782_at	Sstr2	somatostatin receptor 2	1.23	4.73E-05
1369067_at	Nr4a3	nuclear receptor subfamily 4, group A, member 3	1.21	4.43E-05
1377064_at	Dusp6	dual specificity phosphatase 6	1.16	2.64E-05
1373257_at	RGD1307215	similar to protein phosphatase 1, regulatory subunit 1C; thymocyte ARPP	1.08	1.14E-05
1374139_at	Cdr2	cerebellar degeneration-related 2	1.08	5.24E-05
1387087_at	Cebpb	CCAAT/enhancer binding protein (C/EBP), beta	1.08	3.87E-04
1367743_at	Pfkl	phosphofructokinase, liver, B-type	−1.09	1.52E-02
1370922_at	Ctxn	cortexin	−1.12	1.37E-02
1369128_at	Grik5	glutamate receptor, ionotropic, kainate 5	−1.12	1.74E-02
1375149_at	Lrrc4b	leucine rich repeat containing 4B	−1.14	1.02E-02
1368449_at	Centa1	centaurin, alpha 1	−1.17	2.46E-02
1388785_at	Dnalc4	dynein, axonemal, light chain 4	−1.17	4.19E-04
1369792_at	Gpr6	G protein-coupled receptor 6	−1.19	7.57E-05
1390828_at	Npy1r	neuropeptide Y receptor Y1	−1.25	7.65E-05
1367868_at	Adrm1	adhesion regulating molecule 1	−1.33	4.69E-02
1387042_at	Cacnb3	calcium channel, voltage-dependent, beta 3 subunit	−1.38	3.41E-02
1375978_at	Fcho1	FCH domain only 1	−1.41	1.08E-02
1370381_at	Pnrc1	proline rich 2	−1.55	2.64E-05
1368459_at	Gdf10	growth differentiation factor 10	−1.63	1.36E-04

Upregulated genes included many transcription factors, consistent with immediate early gene responses. The set of upregulated genes nonetheless shows some nervous system specificity, including the upregulation of RNAs modulating synaptic function, including Homer1, Arc, Syt4, and Pnoc. Both upregulated and downregulated genes included RNAs involved in metabolism (e.g. sulforedoxin 1 [Srxn1], glutaminase [Gls], phosphofructokinase [Pfkl]). Downregulated genes include receptors and ion channel subunits, such as the voltage-dependent calcium channel beta-3 subunit (Cacnb3), the GluR5 ionotropic glutamate receptor subunit (Grik5), the orphan G-protein coupled receptor Gpr6, and the Y1 neuropeptide Y receptor (Npy1r).

### BDNF-induced genes have different temporal response profiles

**Figure 2:**
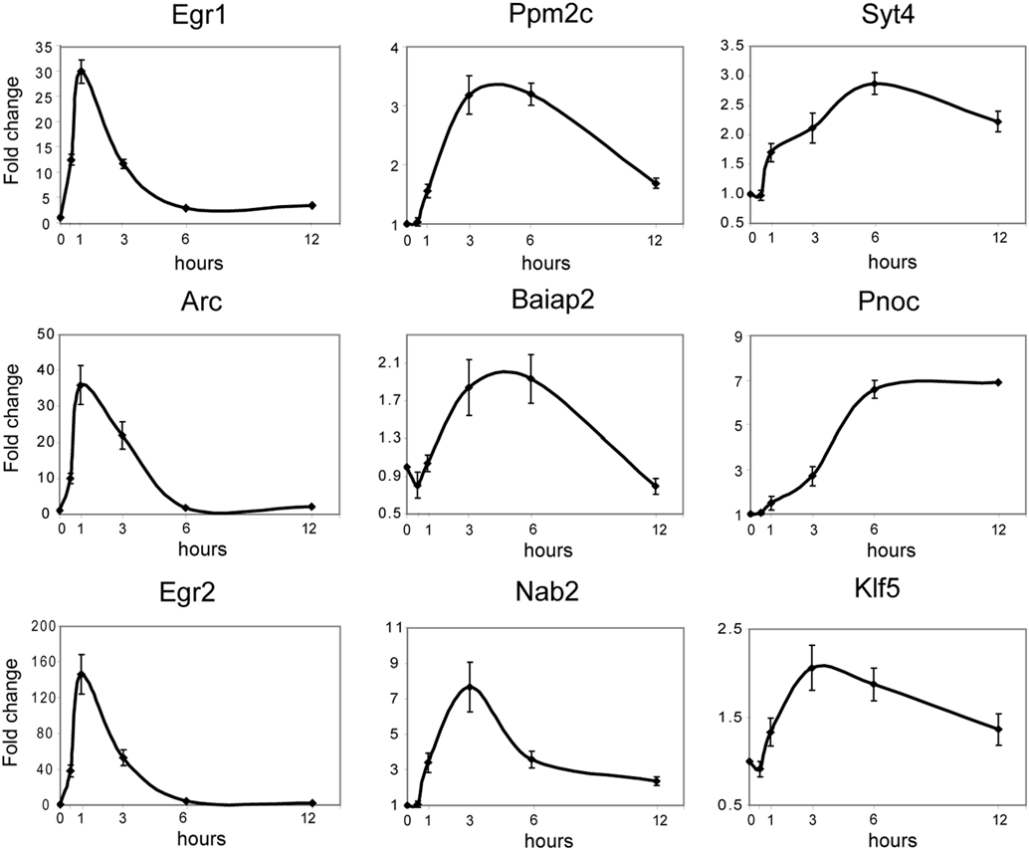
Varying temporal expression profiles of BDNF-mediated gene induction in E16 rat-derived striatal neurons. Transcriptional responses to BDNF show 3 different temporal profiles in cultured striatal neurons. The first group, represented by Egr1, Egr2 and Arc, shows a large and rapid increase within 1 hour and return back to baseline at approximately the 6^th^ hour of treatment. The second set, represented by Ppm2c, Nab2, Klf5 and Baiap2, shows a more gradual and moderate fold increase rising to a peak within 3–6 hours after treatment and decreasing to near baseline levels within 12 hours. The third group, represented by Syt4 and Pnoc, is slower to respond, requiring approximately 6 hours to reach peak levels, which remain elevated through the 12^th^ hour of treatment. X axis represents hours of continuous BDNF treatment; Y axis represents β-actin-normalized fold change; error bars represent SEM for n = 5 biological replicates assayed in triplicate for each condition.

Acute signal-induced gene regulation in the nervous system often has many temporal components [31]. We therefore asked whether BDNF-induced gene expression responses in striatal neurons would have heterogeneous temporal profiles. We assessed selected gene expression responses to BDNF by QPCR after 0.5, 1, 3, 6 and 12 h of BDNF exposure (Fig. 2). Indeed, we were able to discriminate three temporal profiles of gene induction: one showing maximal induction within 1 h and rapidly returning to baseline (despite continued BDNF exposure, *e.g.* Egr1, Egr2 and Arc), one showing less rapid induction (within 3–6 hours) and declining to baseline within 12 hours (e.g. Ppm2c, Nab2, Klf5 and Baiap2), and a third rising slowly to maximum (requiring 6 hours) but showing sustained induction through 12 h of treatment (e.g. Syt4, Pnoc). Genes whose induction shows different temporal profiles may be regulated by different BDNF-induced mechanisms; conversely, genes showing similar induction profiles may be regulated by common mechanisms and potentially subserve related functions (see below).

### Transcriptional responses to BDNF require TrkB and calcium

**Figure 3:**
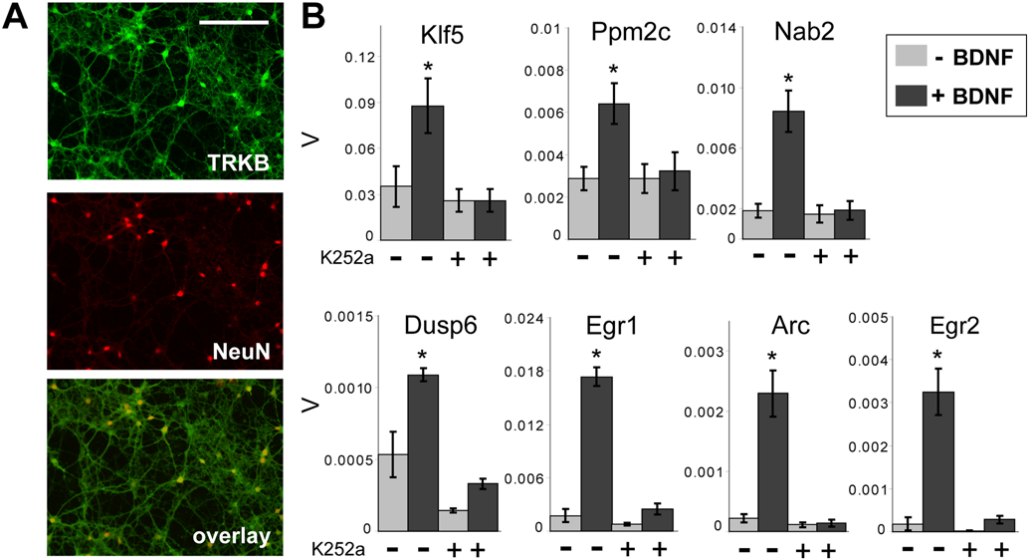
Striatal gene expression responses to BDNF require TrkB. (A) Cultured E16 striatal neurons uniformly express TrkB receptors, as shown by co-immunolabeling for TrkB and the neuronal marker NeuN. Scale bar: 100 µm. (B) The TrkB dependence of BDNF-induced gene expression is demonstrated by blockade of this response by the specific Trk inhibitor K252a (200 nM). 30 min pretreatment of K252a results in near-complete inhibition of BDNF-induced gene expression for all genes tested. Scale of Y axis is β-actin-normalized expression values (V); error bars represent SEM for n = 4 biological replicates assayed in triplicate for each condition. *p<0.03 compared with all other treatments (Student's t-test). K252a also reduced the basal expression of DUSP6.

We next assessed the extent to which striatal neuron gene expression responses to BDNF might be conveyed by its high-affinity receptor TrkB. Immunocytochemical staining of primary cultures for TrkB showed a uniform labeling of all NeuN-positive cells, confirming a constitutive expression of this BDNF receptor in our cultured striatal neurons (Fig. 3A). We then examined the TrkB-dependence of BDNF responses pharmacologically. 30-min pretreatment with the Trk antagonist K252a (200 nM) was sufficient to block BDNF-induced gene expression as compared to vehicle-treated controls (Fig. 3B). These data show that TrkB is the major mediator for acute BDNF-induced effects on striatal gene expression.

**Figure 4:**
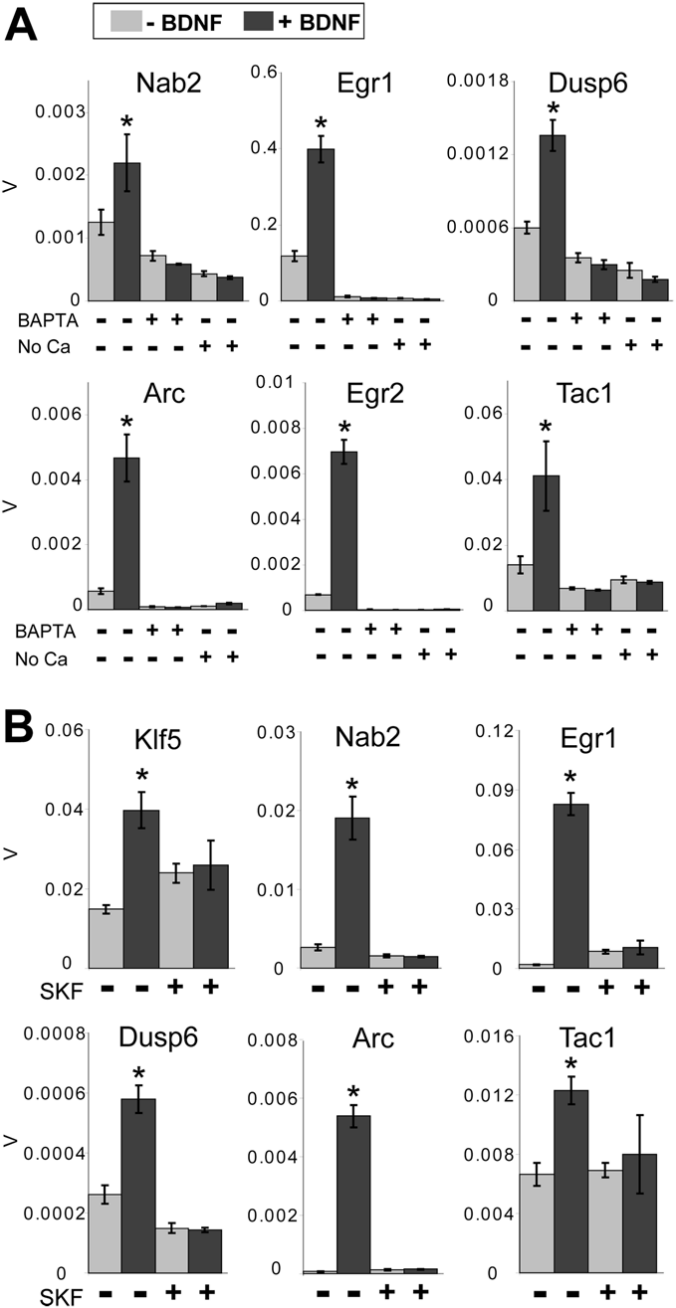
Calcium-dependence of gene expression responses to BDNF in E16 rat primary striatal neurons. (A) Treatment with the cell-permeable calcium chelator BAPTA-AM (100 µM) or omission of calcium from the culture medium completely inhibited BDNF-induced gene expression *p<0.006 compared with all other treatments (Student's t-test). The Y axis represents β-actin-normalized expression values (V); error bars represent SEM for n = 4 biological replicates assayed in triplicate for each condition. (B) The TRPC channel antagonist SKF-96365 hydrochloride (100 µM) also inhibits BDNF-mediated gene expression responses. The Y axis represents β-actin-normalized expression values (V); error bars represent SEM for n = 4 biological replicates assayed in triplicate for each condition. *p<0.002 compared with all other treatments (Student's t-test).

We then assessed whether gene expression responses to BDNF in striatal cells are calcium-dependent. Removal of extracellular calcium or incubation with the intracellular calcium chelator BAPTA-AM for 30 min prior to BDNF stimulation blocked the induction of gene expression responses (Fig. 4A). In order to assess whether TRPC activation contributes to the calcium-mediated effects of BDNF, responses were assessed after 30 min pretreatment with the TRPC antagonist SKF96365 (100 µM) [11]. SKF96365 also blocked BDNF-induced gene expression for all genes tested (Fig. 4B). These results indicate that BDNF-induced striatal gene transcription requires calcium and TRPCs.

### BDNF-mediated MEK/ERK activation is universally required for gene induction while PI3K, CAMK and NOS activation co-regulate induction on a gene-specific basis

**Figure 5:**
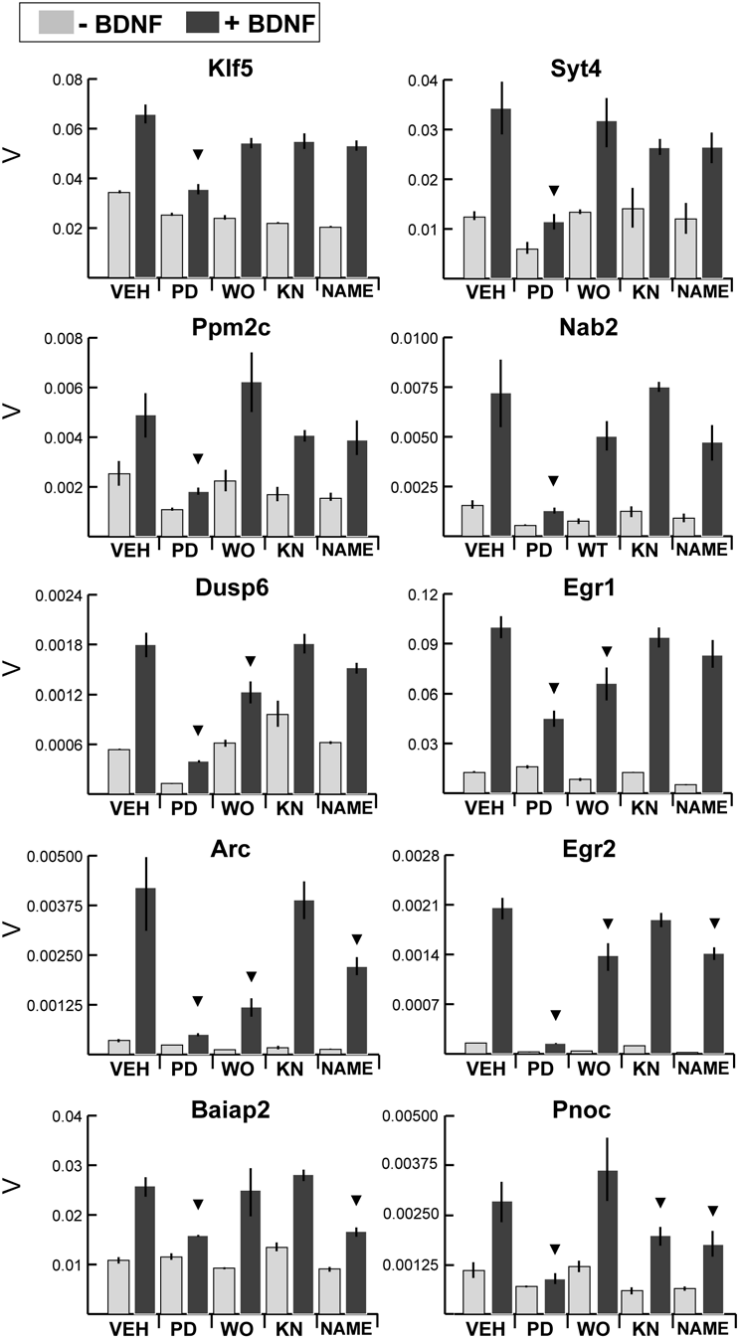
Pharmacological dissection of signaling mechanisms responsible for gene expression responses to BDNF in E16 rat striatal neurons. Y axis represents β-actin normalized expression values (V); error bars represent SEM for n = 4 biological replicates assayed in triplicate for each condition. X axis shows inclusion of vehicle alone (VEH) or inhibitor (PD = MEK inhibitor PD98059 (50 µM), WO = PI3K inhibitor wortmannin (100 nM), KN = CAMK inhibitor KN-93 (5 µM), NAME = NOS inhibitor L-NAME (2 mM). Cutoff criterion for inhibition, indicated by ▾, is a statistically significant [p<0.05 by Student's t-test] diminution of >30% relative to vehicle+BDNF treated expression. BDNF-induced gene expression was inhibited by the specific MEK1/2 inhibitor PD98059 (PD) for all genes tested. The expression of some RNAs also showed sensitivity to PI3K, NOS or CAMK inhibitors. Data demonstrating the efficacious blockade of MEK, PI3K and NOS by these inhibitors under our experimental conditions are presented in Suppl. Fig. 1.

**Figure 6:**
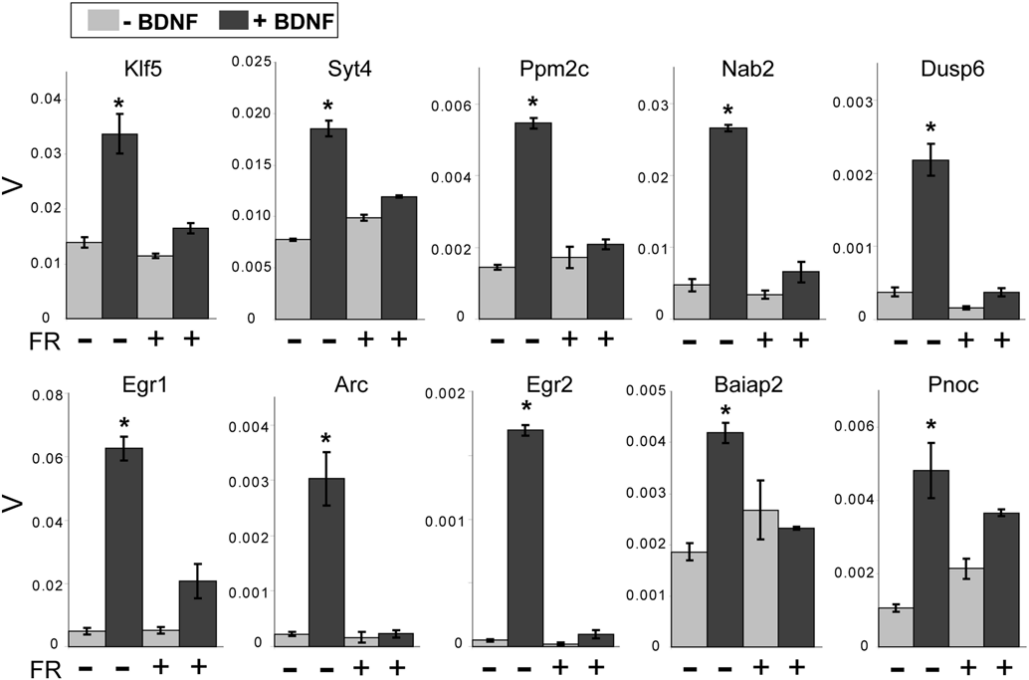
MEK-dependent effects on BDNF-induced gene expression are attributable to ERK. The specific ERK1/2 inhibitor FR180204 (100 µM) exhibited significant blockade of BDNF-induced gene expression in E16 rat ganglionic eminence cultures for all genes tested. Y axis represents β-actin normalized expression values (V) *p<0.05 (Student's t-test) compared to all other conditions. Error bars represent SEM. n = 3–4 biological replicates assayed in triplicate for each condition.

Given that BDNF is known to activate multiple intracellular signaling cascades (Fig. 1), we asked which of these were important for BDNF-mediated gene expression responses in striatal neurons. In order to test the contribution of each pathway, we targeted each by using specific pharmacological inhibitors (as shown in Fig. 1 and Suppl. Fig. S1). For all genes tested, the MEK1/2 inhibitor PD98059 (50 µM) exhibited significant blockade of BDNF-induced expression (Fig. 5). This MEK dependence was further confirmed with a second MEK1/2 inhibitor U0126 (30 µM, Suppl. Fig. S2). MEK-dependent effects of BDNF are largely believed to be mediated through ERK activation [12]. In order to verify the ERK dependence of RNA induction in striatal MSNs, we assessed the effect of the ERK1/2 inhibitor FR180204 (100 µM) on BDNF-induced gene expression (Fig. 6). FR180204, like the MEK inhibitors, resulted in strong, universal blockade of transcriptional responses to BDNF for all genes tested. We thus conclude that MEK and ERK activation comprises a major central pathway of short-term BDNF-dependent transcriptional responses in striatal neurons.

In contrast to the universal effects of MEK and ERK blockade, inhibitors of PI3K (wortmannin, 100 nM), NOS (L-NAME, 2 mM), and CAMK (KN-93, 5 µM) showed gene-specific actions. Whereas the BDNF-regulated expression of Klf5, Syt4, Nab2 and Ppm2c was blocked only by MEK inhibition, other genes showed modulation by two or more pathways. BDNF-mediated induction of Egr1 and Dusp6 were blocked by either MEK or PI3K inhibition. Egr2 and Arc induction was diminished by MEK, PI3K or NOS inhibition. Baiap2 induction was reduced by inhibitors of either MEK or NOS. BDNF stimulation of Pnoc expression was inhibited by MEK, CAMK, or NOS inhibition. These results indicate gene-specific roles for PI3K, NOS, and CAMK in the modulation of striatal BDNF responses in concert with necessary activation of MEK and ERK.

### BDNF-mediated activation of nuclear transcription factors Elk-1 and CREB is MEK-dependent

**Figure 7:**
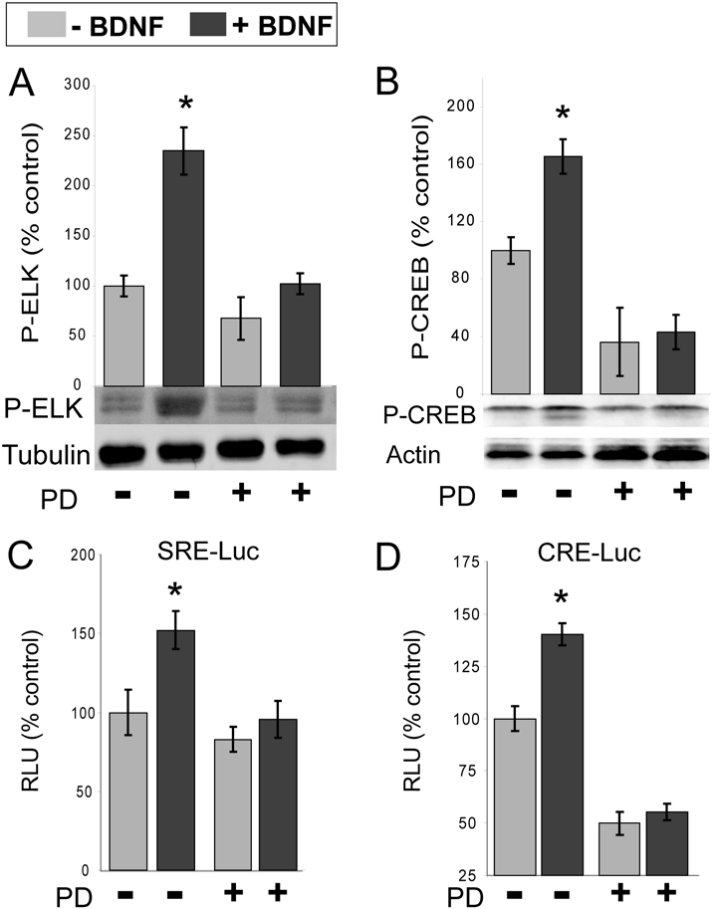
BDNF-induced activation of ELK and CREB is MEK-dependent. (A,B) Stimulation of E16 rat ganglionic eminence cultures with BDNF (50 ng/ml) for 15 min, results in increased phosphorylation of ELK1 at Ser 383 and CREB at Ser133. 30 min pretreatment with the specific MEK1/2 inhibitor PD98059 blocked BDNF-induced phosphorylation. Phosphorylation of ELK and CREB is shown by representative immunoblots, with quantitation summarized in the bar graphs. Error bars represent SEM for n = 3–4. *p<0.05 compared to other conditions (Student's t-test). Representative immunoblot images are shown below the bar graphs. (C) A SRE-luciferase promoter reporter assay for Elk1/TCF-mediated transcriptional activity (see Materials and Methods) showed increased SRE-dependent transcriptional activity after BDNF treatment (50 ng/ml for 90 min). SRE-dependent gene expression was blocked by 30 min pretreatment with the specific MEK1/2 inhibitor PD98059 (50 µM). Y axis represents percentage luminescence ratio of SRE signal to normalization control PGK promoter signal; Error bars represent SEM for n = 3–4. *p<0.05 compared to other conditions (Student's t-test). (D) A CRE-luciferase promoter reporter assay for CREB activation (see Experimental Procedures) also showed increased CRE-dependent transcriptional activity after BDNF treatment (50 ng/ml for 90 min). CRE-dependent gene expression was blocked by 30 min pretreatment with the specific MEK1/2 inhibitor PD98059 (50 µM). Y axis represents relative luminescence units (RLU); Error bars represent SEM for n = 5. *p<0.05 compared to other conditions (Student's t-test).

Previous studies have shown that several DNA-binding transcription factors are phosphorylated in a BDNF-dependent manner and that their activities are required for BDNF-induced gene expression [14], [32]. To assess whether the phosphorylation and activation of such factors in striatal neurons is dependent on MEK and ERK, we tested whether these effects were sensitive to pharmacologic inhibition of the MAPK cascade. The MEK1/2 inhibitor PD98059 was able to block the BDNF-induced phosphorylation of the SRF-associated TCF protein Elk-1 (Fig. 7A) and the CRE-binding protein CREB (Fig. 7B). Enhancer-reporter constructs also show a BDNF-induced regulation of SRE- and CRE-associated transcriptional activity which is sensitive to pharmacologic inhibition of the MAPK cascade, as measured using the activation of SRE- and CRE-driven luciferase expression in striatal neurons (Fig. 7C,D). These results suggest that MEK/ERK activation is important for both SRF/TCF- and CREB-dependent gene expression initiated by BDNF in striatal cells.

### MEK is also required for BDNF-induced mRNA responses in adult striatal neurons

**Figure 8:**
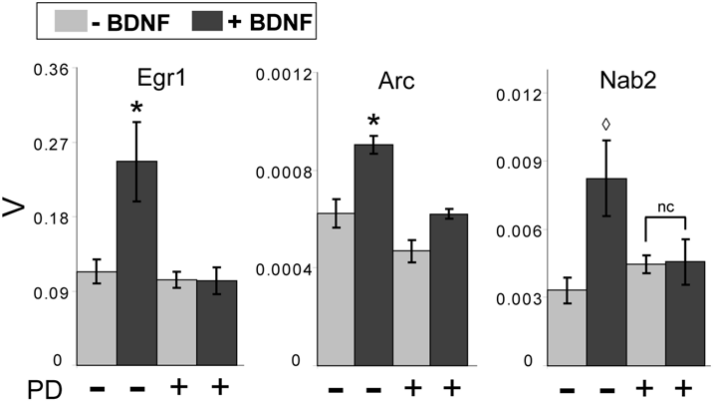
Adult striatal neurons show similar MEK dependence of BDNF-induced gene expression responses. Neurons acutely dissociated from 30-day-old rat striata were exposed to 50 ng/ml BDNF with or without the specific MEK inhibitor PD98059 (50 µM). BDNF-induced expression of Egr1, Arc and Nab2 RNAs is blocked by PD98059, indicating that MAPK is a required mediator of BDNF-induced gene expression in the adult brain. Y axis scale represents β-actin-normalized expression values (V); error bars represent SEM. Egr1 and Arc show significantly higher expression in BDNF-treated cells than in vehicle-treated, PD98059-treated or PD98059+BDNF-treated cells *p<0.05 (Student's t-test). Nab2 expression is significantly induced by BDNF ◊ p<0.05 (Student's t-test) and this induction blocked by PD98059 treatment (“nc” indicates no significant change in Nab expression in PD98059+BDNF-treated versus PD98059-treated cells [p>0.05 by Student's t-test]). n = 3–4 biological replicates assayed in triplicate for each condition.

Whereas the above studies were performed in cells cultured from embryonic rat brain, we wished to explore whether ERK also played a major role in the BDNF-mediated regulation of gene expression in the mature striatum. We thus assessed the effects of BDNF on striatal neurons dissociated from adult rat brain (at postnatal day 30). For a high proportion of genes tested, the dissociation procedure itself increased expression such that the detection of BDNF-related induction could no longer be measured. Nonetheless, for all three genes whose expression remained BDNF-responsive (Egr1, Arc, and Nab2), BDNF-mediated induction was MEK-dependent (Fig. 8). These data indicate that MEK/ERK activation is also an important mediator of striatal gene regulation in the adult brain.

### De novo MEK/ERK-dependent transcription is important for the BDNF-regulated structural plasticity of striatal neurons

**Figure 9:**
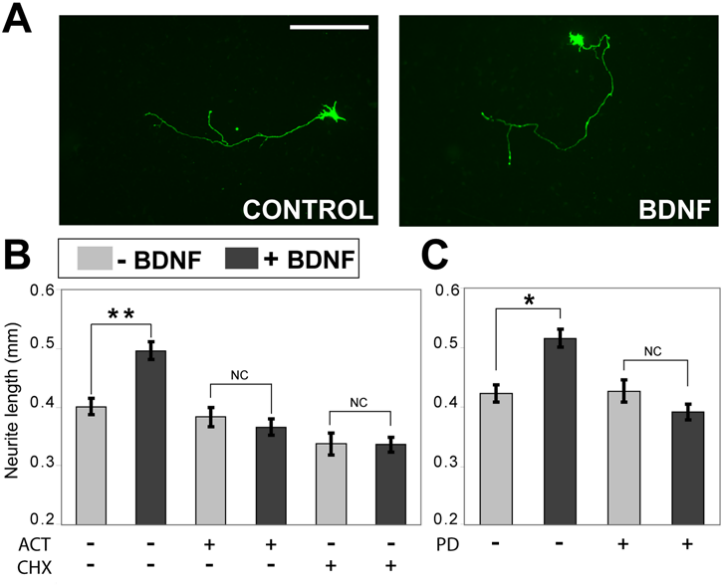
MAPK and new transcription are required for BDNF-induced striatal neurite outgrowth. (A) Examples of E16 rat ganglionic eminence-derived neurons transfected with EGFP vector and either stimulated with BDNF for 24 h or untreated. Scale bar: 0.5 mm. (B,C) Length of longest neurite of individual striatal cells treated with BDNF for 24 h, presented as mean±SEM of neurite length in millimeters). **p<0.001, *p<0.05 compared to untreated cells (Student's t-test). Treatment with cycloheximide (0.5 ug/ml) or actinomycin D (2 ug/ml) starting 30 min prior to BDNF exposure prevented BDNF-induced neurite outgrowth (B). Numbers of cells analyzed (panel B): Control = 150, BDNF = 160, ACT = 85, ACT+BDNF = 93, CHX = 60, CHX+BDNF = 65. Treatment with PD98059 also prevented BDNF-induced neurite outgrowth (C). Numbers of cells analyzed (panel C): Control = 65, BDNF = 90, PD = 50, PD+BDNF = 63.

Previous studies have suggested that BDNF may be an important mediator of structural aspects of neuronal plasticity [33], [34], [35]. In the context of the present results, we wished to explore whether BDNF-induced regulation of striatal gene expression might contribute to such an effect in striatal cells. To address this question, we first assessed changes in neurite morphology in cultured striatal neurons following exposure to BDNF. Whereas we observed no apparent BDNF-induced neuritic spine formation in our cultured striatal cells (data not shown), we observed a significant BDNF-dependent increase in axon length (as assessed by length of longest neuronal process; Fig. 9B,C). Further testing showed that this BDNF-induced change in cellular morphology was dependent on *de novo* gene and protein expression, as demonstrated by its sensitivity to transcriptional and translational inhibitors (actinomycin D and cyclohexamide, respectively Fig. 9B). Moreover, the neurite outgrowth-promoting effect of BDNF was also dependent on MEK activation, as shown by its complete blockade by PD98059 (Fig. 9C). Therefore, we provide evidence that important structural plasticity effects of BDNF in striatal neurons are mediated by MEK-dependent signaling pathways involving transcriptional regulation.

## Discussion

In this study, we elucidate mechanisms underlying BDNF-induced gene expression and show their importance for the molecular and structural plasticity of striatal neurons. Although BDNF has been heavily implicated as an important regulator of striatal function, a comprehensive genome-wide study of BDNF's transcriptomic effects in these cells has not previously been reported. We therefore performed a systematic assessment of early-phase gene expression responses to BDNF in striatal neurons using microarray analysis and subsequently studied the signaling pathways regulating BDNF responses using pharmacologic manipulations followed by QRT-PCR. Our chosen approach to block these signaling pathways with small molecules avoids the potential for non-BDNF-dependent effects of genetic manipulation and allows tight temporal regulation of the inhibition. Although the MEK inhibitors used for our study could potentially inhibit ERK5, the final conclusion that a MEK/ERK signaling pathway underlies BDNF's effects remains unchanged. Moreover, the specific implication of MEK1/2 and ERK1/2 are further supported by the observation of the same effects using a selective ERK1/2 inhibitor.

These analyses indicate that MEK and ERK serve as gatekeepers for BDNF-induced gene expression in striatal cells. We also show that BDNF-mediated transcriptional activation in striatal cells requires TrkB receptors and intracellular and extracellular calcium. Moreover, we observe that PI3K, CAMK and NOS modulate BDNF responses in a gene-specific manner in these GABAergic neurons.

This first transcriptome-wide analysis of the proximal gene-induction effects of BDNF in striatal cells comprises an important advance which complements previous studies of individual gene responses and complex phenotypes. Moreover, the evidence for a heavy reliance of BDNF-mediated gene expression responses on MEK/ERK and TRPC discloses important new aspects of the mechanisms underlying BDNF-associated neuronal plasticity. While the extensive analysis of gene expression responses in striatal cells is new, we will elaborate in the following paragraphs how the present results compare with previous data collected mainly in non-striatal cell types.

The specific genes shown to be BDNF-responsive in striatal neurons are generally concordant with previous findings in other neural cells and tissues [19], [30]. The heterogeneous timing of BDNF-initiated transcriptional responses into several temporal phases is also consistent with previous studies which have shown several waves of BDNF-induced gene expression in cultured cerebellar granule cells [30]. Where differences are observed between the specific gene responses measured in our system and those of previous studies, some of these are undoubtedly biological (e.g. due to differences neuronal type), while others may be technical (e.g. due to the use of different microarrays). However, we interpret the fact that specific and distinct nervous system-related mRNAs are induced in striatal cells (both in this and previous studies such as [36]) to suggest the former, *i.e.* to support that gene induction by BDNF exhibits some cell-type specificity. In either case, our results provide a basis for further testing of neuronal-type-specific BDNF effects.

Our results indicate that BDNF-induced gene expression is important for its structural remodeling of striatal neurons, as assessed by its neurite-outgrowth-promoting activity. Although we have not examined the contributions of individual BDNF-induced genes to neurite outgrowth in the present study, previous data suggests that effectors might include Pnoc [30], Egrs [37], Vasp [38], Cdkn1a/p21, Arc [39], PDGF [40] and IGF1 [41].

Mechanisms of short-term BDNF-mediated changes in gene expression have also been explored previously in various non-striatal primary cell types. Previous studies in periodontal cementoblasts and in primary hippocampal cultures also support that short-term BDNF-induced transcriptional activation relies heavily on MEK but not PI3K signaling [19], [42]. Studies in TrkB mutant mice, however, indicated that the MEK/ERK pathway was less important for BDNF-induced transcriptional regulation than calcium-dependent mechanisms including CAMK [43]. Studies examining the regulation of individual striatal genes have previously ascribed an important role to PI3K [36]. While we examined the expression of different RNAs, our findings are consistent with these previous data in the respect that we also detect significant effects of the PI3K inhibitor wortmannin in the BDNF-mediated regulation of certain genes in primary striatal neurons.

Our results demonstrate requirements of extracellular calcium and TRPC channels in BDNF responses, a perspective also supported by other recent studies [44], [45]. To our knowledge, however, this is the first report to demonstrate TRPC dependence of the BDNF-mediated regulation of specific genes. Beyond CAMK-dependent phosphorylation of the transcriptional activator CREB, other known calcium-dependent intracellular mechanisms of BDNF-regulated gene expression include regulation of chromatin structure via NO-mediated nitrosylation of histone deacetylase 2 [16]. Although other downstream calcium-dependent mechanisms have not specifically been addressed in the present study, our results suggest that CAMK and NOS comprise modulatory, rather than universal, regulatory mechanisms of BDNF-mediated gene induction in striatal cells. However, the major calcium-dependence of BDNF-mediated gene induction observed in our experiments indicates that other calcium-dependent mechanisms are also important for striatal gene expression responses to BDNF. One pathway of potential cross-talk between calcium and MEK/ERK in striatal neurons is the known positive effect of calcium-calmodulin on raf activation [46]. Nonetheless, a deeper understanding of combinatorial (or synergistic) effects of various arms of the BDNF signaling cascade may be essential to deciphering new key aspects of gene regulation via BDNF and TrkB in striatal cells.

The present study focuses on early short-term gene expression effects of BDNF. However, it will also be important to determine how BDNF regulates long-term striatal gene expression on a genome-wide scale, and through which molecular pathways. Previous evidence shows that BDNF is a major regulator of striatal development and the specification of medium spiny striatal projection cells [4], including the regulation of MSN-enriched genes such as DARPP-32 [36], [47]. Moreover, in addition to phenotype specification and plasticity, it will be important to ascribe neuroprotective activities of BDNF in striatal cells to particular gene expression effects.

Another aspect of BDNF's modulation of striatal transcription which is uncovered by the present experiments is the negative regulation of the expression of a considerable number of genes. Although we are not able to ascribe a specific function to this set of downregulated RNAs at present, it is intriguing to note that they include RNAs encoding signaling and intracellular transport proteins that could be important for fine-tuning neuronal input.

In addition to the regulation of striatal neuron differentiation, corticostriatal BDNF signaling has been implicated in the etiology and treatment of neurodegenerative and neuropsychiatric disorders including drug addiction [48], depression [49], [50], schizophrenia [51], and Huntington's disease (HD) [52], [53], [54], [55]. Of particular interest to us is the finding that the striatal gene expression profile of forebrain-specific conditional BDNF null-mutant mice exhibits a strong relationship to that of human HD caudate [56]. Although HD-related striatal cell loss [57], abnormalities in BDNF gene transcription [58] or BDNF transport [59] could be sufficient to explain this similarity, it will also be important to explore whether postsynaptic striatal responses to BDNF may contribute to the pathophysiology of HD. The present results provide a basis for further assessment of abnormalities of BDNF-dependent gene expression in striatal cells and its potential involvement in HD or clinically relevant human brain disorders.

## Supporting Information

Figure S1 BDNF-mediated activation of ERK, PI3K and NOS in E16 rat ganglionic eminence cultures and their effective blockade with pharmacologic inhibitors. (A) Stimulation with BDNF (50 ng/ml) increases in the phosphorylation of Akt (at Ser473) within 15 min. 30-min pretreatment with the PI3K inhibitor wortmannin (100 nM, WO) prevented BDNF induced Akt phosphorylation, whereas the MEK1/2 inhibitors PD98059 (50 µM, PD) or U0126 (30 µM, U) had no effect. (B) Stimulation with BDNF (50 ng/ml) increased the phosphorylation of Erk1/2 (Thr202/Tyr204) within 15 min. 30-min pretreatment with MEK1/2 inhibitor PD98059 (50 µM) or U0126 (30 µM) prevented BDNF-induced ERK phosphorylation, whereas the PI3K inhibitors wortmannin (100 nM, WO) had no effect. (C) BDNF-mediated activation of NOS was detected with DAF-FM DA after stimulation with BDNF (50 ng/ml for 30 min). 30 min pretreatment with NOS inhibitor L-NAME (2 mM) prevented NO formation *p<0.00006 (Student's t-test). Y axis represents the mean fluorescence intensity of the NO signal calculated as described in Experimental Procedures. Error bars represent SEM for n = 16–21.
(0.34 MB TIF)

Figure S2 MEK1/2 inhibitor U0126 also inhibits BDNF-induced gene expression. The specific MEK1/2 inhibitor U0126 (30 µM) exhibited significant blockade of BDNF induced gene expression in E16 rat ganglionic eminence cultures for all genes tested. Y axis scale represents β-actin normalized expression value (V); treatment with BDNF alone induces significantly higher expression than all other conditions *p<0.02 (Student's t-test). Error bars represent SEM for n = 4 biological replicates assayed in triplicate for each condition.
(0.24 MB TIF)

Table S1 RNA changes in striatal neurons after 3 h treatment with BDNF. Data for all microarray probesets meeting criteria of FDR p<0.05 are shown (see text).
(1.49 MB XLS)
