# Supplementary figures and images for: Short-Term Striatal Gene Expression Responses to Brain-Derived Neurotrophic Factor Are Dependent on MEK and ERK Activation
Source: PLoS One. 2009 Apr 23;4(4):e5292. doi: 10.1371/journal.pone.0005292 (PMC2669182; doi:10.1371/journal.pone.0005292)

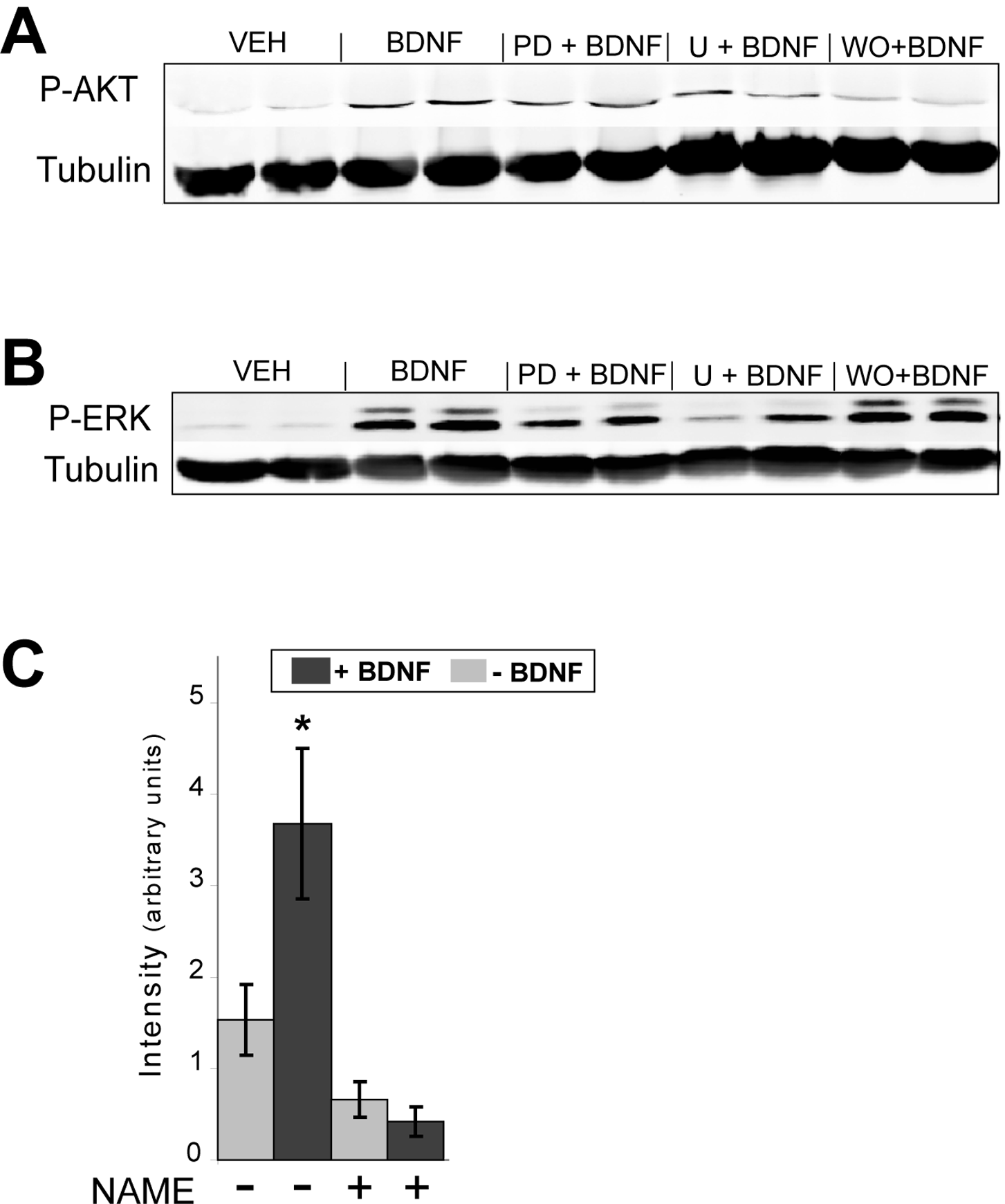

Supplement: Figure S1 — BDNF-mediated activation of ERK, PI3K and NOS in E16 rat ganglionic eminence cultures and their effective blockade with pharmacologic inhibitors. (A) Stimulation with BDNF (50 ng/ml) increases in the phosphorylation of Akt (at Ser473) within 15 min. 30-min pretreatment with the PI3K inhibitor wortmannin (100 nM, WO) prevented BDNF induced Akt phosphorylation, whereas the MEK1/2 inhibitors PD98059 (50 µM, PD) or U0126 (30 µM, U) had no effect. (B) Stimulation with BDNF (50 ng/ml) increased the phosphorylation of Erk1/2 (Thr202/Tyr204) within 15 min. 30-min pretreatment with MEK1/2 inhibitor PD98059 (50 µM) or U0126 (30 µM) prevented BDNF-induced ERK phosphorylation, whereas the PI3K inhibitors wortmannin (100 nM, WO) had no effect. (C) BDNF-mediated activation of NOS was detected with DAF-FM DA after stimulation with BDNF (50 ng/ml for 30 min). 30 min pretreatment with NOS inhibitor L-NAME (2 mM) prevented NO formation *p<0.00006 (Student's t-test). Y axis represents the mean fluorescence intensity of the NO signal calculated as described in Experimental Procedures. Error bars represent SEM for n = 16–21. (0.34 MB TIF) [file pone.0005292.s001.tif]

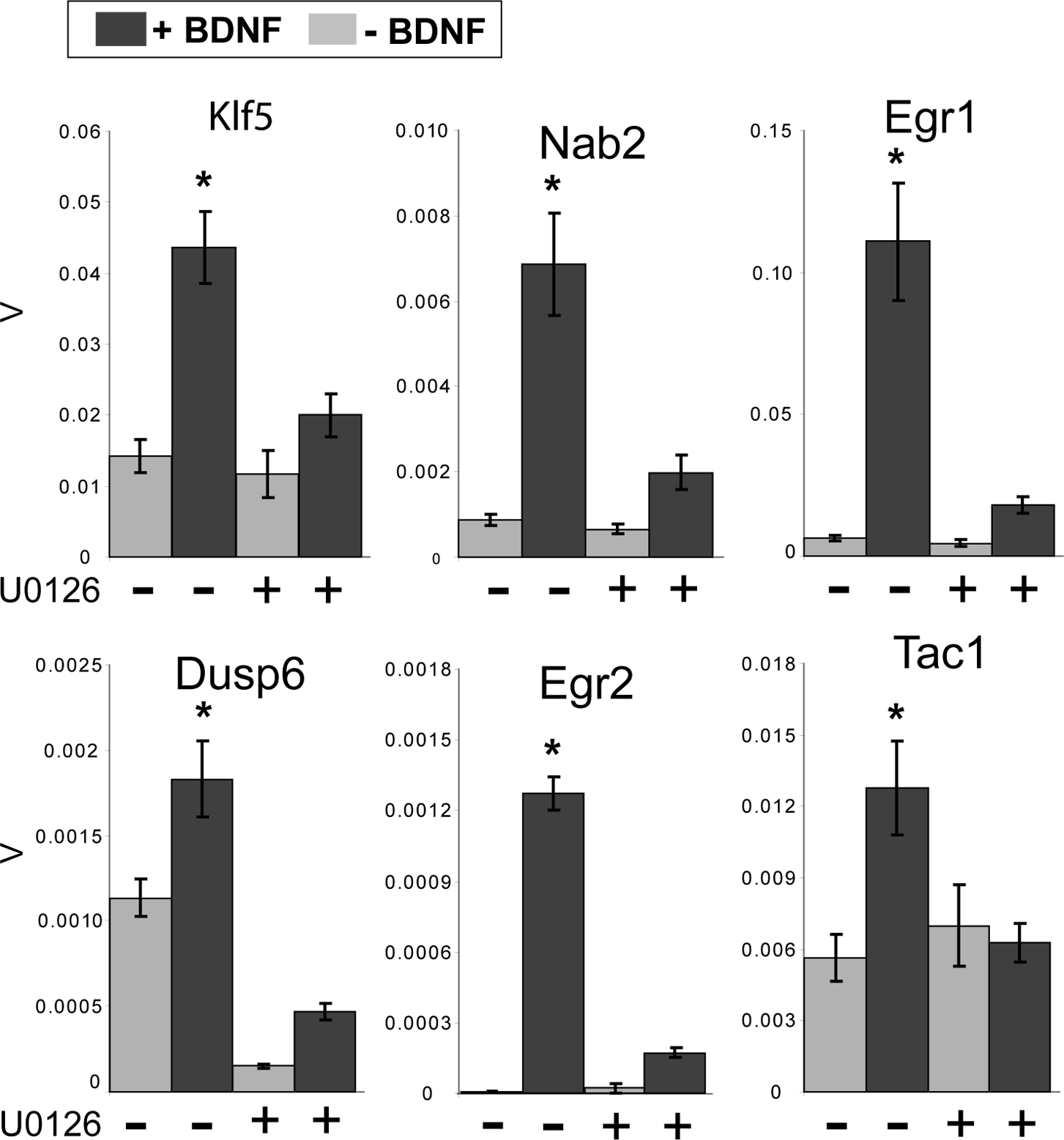

Supplement: Figure S2 — MEK1/2 inhibitor U0126 also inhibits BDNF-induced gene expression. The specific MEK1/2 inhibitor U0126 (30 µM) exhibited significant blockade of BDNF induced gene expression in E16 rat ganglionic eminence cultures for all genes tested. Y axis scale represents β-actin normalized expression value (V); treatment with BDNF alone induces significantly higher expression than all other conditions *p<0.02 (Student's t-test). Error bars represent SEM for n = 4 biological replicates assayed in triplicate for each condition. (0.24 MB TIF) [file pone.0005292.s002.tif]
